# Prolonged Respiratory Alkalosis Induced by Caffeine at Subtoxic Serum Concentrations

**DOI:** 10.7759/cureus.85591

**Published:** 2025-06-09

**Authors:** Fumiya Inoue, Yuji Okazaki, Toshihisa Ichiba, Takuyo Chiba, Akira Namera

**Affiliations:** 1 Department of Emergency Medicine, Hiroshima City Hiroshima Citizens Hospital, Hiroshima, JPN; 2 Department of Emergency Medicine, International University of Health and Welfare, Chiba, JPN; 3 Department of Forensic Medicine, Hiroshima University, Hiroshima, JPN

**Keywords:** caffeine toxicity, hyperventilation, respiratory alkalosis, serum concentration, tachypnea

## Abstract

Respiratory alkalosis due to hyperventilation is a common acid-base disturbance in emergency care settings. While often linked to anxiety or panic attacks, drug-induced causes must also be considered. Caffeine, a widely consumed methylxanthine, is known to induce respiratory alkalosis due to tachypnea in severe overdoses. However, the effects of caffeine at subtoxic serum concentrations are not well characterized, and the possibility of caffeine-induced respiratory alkalosis in the absence of tachypnea is not usually recognized.

A 47-year-old underweight female patient with a history of depression presented with general weakness. Her vital signs were stable, and her respiratory rate was 15 breaths per minute. Physical examination revealed carpopedal spasms and extremity weakness. Laboratory tests showed hypokalemia and a venous blood gas with a pH of 7.57 and partial pressure of carbon dioxide (pCO₂) of 27.4 mmHg, consistent with respiratory alkalosis. Despite intravenous electrolyte replacement, respiratory alkalosis persisted for over 20 hours without evidence of tachypnea. Serum caffeine concentration measured by liquid chromatography-mass spectrometry was 13.6 µg/mL at admission and 6.6 µg/mL at 36 hours post-presentation. No other toxic agents were detected. She recovered with supportive care 36 hours after admission. The final diagnosis was caffeine-induced respiratory alkalosis, occurring in the absence of tachypnea and at subtoxic serum caffeine concentrations.

This case illustrates that caffeine can provoke respiratory alkalosis through increased tidal volume, even at subtoxic serum concentrations and in the absence of tachypnea. Clinicians should consider caffeine toxicity in the differential diagnosis of unexplained respiratory alkalosis and obtain a detailed history, including dietary and supplement use.

## Introduction

Respiratory alkalosis due to hyperventilation is a common acid-base disorder in the emergency department (ED) [1]. While this condition is often associated with psychiatric symptoms such as anxiety or panic attacks, it may also be caused by serious underlying conditions, including sepsis, pulmonary embolism, or cardiovascular disorders [2]. Thus, identifying the underlying cause of hyperventilation is clinically crucial in emergency clinical practice.

Drug-induced hyperventilation is an important cause to consider in the differential diagnosis of respiratory alkalosis [2]. Salicylate poisoning is a well-known cause, but other pharmacological agents such as methylxanthines, beta (β)-adrenergic agonists, and progesterone have also been implicated [2, 3]. Caffeine, the most widely consumed methylxanthine, may provoke respiratory alkalosis due to tachypnea in cases of acute severe overdose [4, 5]. However, the respiratory effects of non-severe caffeine poisoning are rarely described, and there are no previous reports clearly describing the association between serum caffeine concentrations and the development of respiratory alkalosis.

We present a case of prolonged respiratory alkalosis with normal respiratory rate due to caffeine poisoning despite serum caffeine levels being below toxic concentrations.

## Case presentation

A 47-year-old female patient presented to our ED with general weakness lasting for 12 hours. She had depression that was treated with two tablets of 25 mg sertraline hydrochloride, two tablets of 10 mg escitalopram oxalate, three tablets of 5 mg bromazepam, and two tablets of 5 mg zolpidem tartrate daily. She appeared underweight (body mass index 17.3 kg/m²), raising concern about possible eating disorders. She continued to be a picky eater, consuming only spaghetti, totaling approximately 400 kcal daily. On arrival, her vital signs were as follows: Glasgow Coma Scale of 15 (E4, V5, M6), body temperature of 37.6°C, heart rate of 108 beats per minute with sinus rhythm, blood pressure of 120/87 mmHg, oxygen saturation of 99% on room air, and a respiratory rate of 15 breaths per minute. Physical examination showed carpopedal spasm and weakness of the extremities. Blood examinations showed hypokalemia, hypomagnesemia, and hypophosphatemia with subclinical hypothyroidism. In addition, venous blood gas analysis revealed acute respiratory alkalosis (Table 1).

**Table 1:** Laboratory examinations pCO_2_: partial pressure of carbon dioxide; HCO3: bicarbonate; ALT: alanine transaminase; AST: aspartate transferase; LD: lactate dehydrogenase; TSH: thyroid-stimulating hormone

Parameters	Hospital admission	Reference range
Venous pH	7.57	7.31 - 7.41
Venous pCO_2_	27.4	35 - 45 mmHg
Venous HCO3	25.2	22 - 46 mmol/L
Venous lactate acid	9.4	0.5 - 1.6 mmol/L
White blood counts	7000	3.3 - 8.6 x 10^3^/μL
Hemoglobin	13.0	13.7 - 16.8 g/dL
Platelet	34.6	15.8 - 34.8 x 10^4^/μL
AST	68	13 - 30 U/L
ALT	42	10 - 42 U/L
LD	216	124 - 222 U/L
Blood urea nitrogen	2	8 - 20 mg/dL
Creatinine	0.71	0.65 - 1.07 mg/dL
Creatinine kinase	310	59 - 248 U/L
C-reactive protein	0.026	< 0.14 mg/dL
Sodium	137.9	138 - 145 mmol/L
Potassium	2.8	3.6 - 4.8 mmol/L
Chloride	94.9	101 - 108 mmol/L
Magnesium	1.0	1.9 - 2.5 mg/dL
Phosphate	0.5	2.7 - 4.6 mg/dL
Glucose	93	73 - 107 mg/dL
TSH	13.9	0.61 - 4.23 mIU/L
Free T4	1.06	0.9 - 1.7 ng/dL

**Figure 1:**
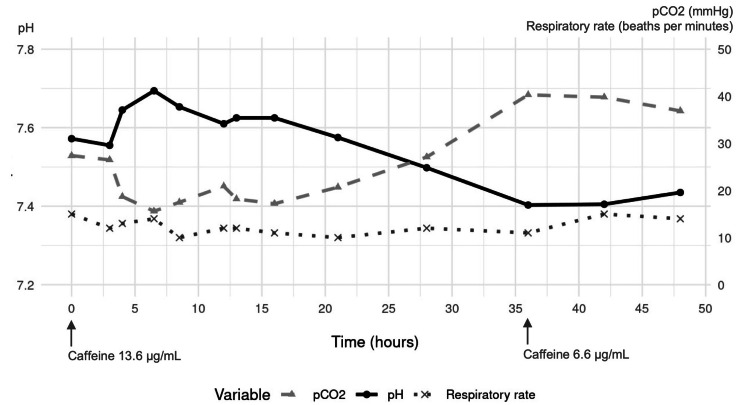
Time course of pH, pco₂, and respiratory rate following admission and changes in caffeine levels pCO_2_: partial pressure of carbon dioxide

A 12-lead electrocardiogram revealed a prolonged QT interval, and transthoracic echocardiography was normal. She was admitted with a diagnosis of hypokalemic paralysis, but the cause of respiratory alkalosis remained unclear. During the first 10 hours of admission, 80 mEq of potassium chloride was intravenously administered along with magnesium and phosphate supplementation. However, respiratory alkalosis with a normal respiratory rate persisted (Figure 1).

With continued treatments, both conditions gradually improved after 20 hours and resolved completely by 36 hours. She was discharged on day 4 without recurrence of respiratory alkalosis due to hyperventilation.

In the following days, testing with a high-performance liquid chromatograph/tandem mass spectrometer (1260 Infinity Liquid Chromatography System and 6420 Triple Quad Mass Spectrometer (Agilent Technologies, Palo Alto, CA, USA)) revealed a serum caffeine concentration of 13.6 µg/mL at admission and 6.6 µg/mL at 36 hours after admission. Serum salicylic acid and other causative drugs, including other methylxanthines, were not detected. In addition, serum concentrations of sertraline, escitalopram, bromazepam, and zolpidem were at therapeutic levels at ED admission. Despite detailed questioning, the exact amount of caffeine consumed could not be determined. Finally, we made a diagnosis of caffeine-induced respiratory alkalosis, as potential causes, including hypoxemia, cardiopulmonary disease, central nervous system disorders, medications, and inflammatory diseases, were excluded based on the initial assessment and her clinical course. However, our patient refused outpatient follow-up for caffeine intake reduction.

## Discussion

The course of this patient highlighted two important clinical issues: 1) caffeine poisoning may present with respiratory alkalosis even in the absence of tachypnea, and 2) respiratory alkalosis can persist unexpectedly even when serum caffeine concentrations are below toxic serum concentrations.

Respiratory alkalosis may be a notable but overlooked feature in caffeine poisoning, especially when compared to other manifestations of toxicity such as vomiting, tachycardia, and hypokalemia. Caffeine stimulates the central nervous system and activates the medullary respiratory center [6]. In cases of severe caffeine poisoning, this can result in tachypnea [4-6]. However, experimental studies have demonstrated that caffeine can increase tidal volume and prolong the inspiratory phase, regardless of respiratory rate [7]. This respiratory effect has been utilized in clinical practice, such as in the treatment of apnea of prematurity, without necessarily elevating the respiratory rate [8, 9]. In our case, respiratory alkalosis was most likely driven by an increased tidal volume, given that the patient’s respiratory rate remained consistently below 20 breaths per minute throughout. Given the lack of prominent tachypnea or other typical signs of caffeine toxicity, we might have overlooked the cause of respiratory alkalosis if serum caffeine levels had not been measured. Clinicians may recognize this atypical presentation of caffeine poisoning and consider it in the differential diagnosis of unexplained respiratory alkalosis, even in the absence of tachypnea, in emergency settings.

Another notable finding was the persistence of respiratory alkalosis despite serum caffeine concentrations falling below the toxic range. The toxic threshold for acute caffeine poisoning is generally considered to be 15-20 μg/mL [10]. At serum concentrations above this level, symptoms such as palpitations, nausea, tremor, and paresthesia typically appear [11]. However, there are limited reports describing the specific serum caffeine concentrations at which respiratory alkalosis occurs. This may be due to variations in the underlying mechanism and whether the alkalosis is driven predominantly by tachypnea or increased tidal volume. The therapeutic range for apnea of prematurity is typically 8-25 μg/mL [8, 12], and even in adults, respiratory alkalosis associated with increased tidal volume may occur at concentrations within or even below this range. In our case, respiratory alkalosis persisted until the serum caffeine concentration decreased to 6.6 μg/mL. This suggests that stimulation of the respiratory center by caffeine may lead to increased tidal volume in adults at concentrations similar to therapeutic concentrations used in neonates. Furthermore, the observed physiological effects at subtoxic concentrations may have been influenced by our patient being underweight. Previous studies have suggested that underweight individuals or those with malnutrition may exhibit increased sensitivity to caffeine, potentially explaining the clinical manifestations despite relatively modest serum levels [13].

## Conclusions

Given the growing popularity of caffeine-containing products and the lack of awareness surrounding habitual consumption, prolonged respiratory alkalosis due to caffeine poisoning should be emphasized due to potential complications such as hypokalemia-induced life-threatening arrhythmias. Especially in patients with unexplained respiratory alkalosis, clinicians should consider caffeine toxicity and obtain a detailed history, including dietary and supplement use.
